# The Impact of the Addition of Compatibilizers on Poly (lactic acid) (PLA) Properties after Extrusion Process

**DOI:** 10.3390/polym12112688

**Published:** 2020-11-14

**Authors:** F.A.M.M. Gonçalves, Sandra M. A. Cruz, Jorge F. J. Coelho, Arménio C. Serra

**Affiliations:** 1 CIEPQPF, Department of Chemical Engineering, University of Coimbra, 3030-790 Coimbra, Portugal; filipaalmeidamartins@gmail.com; 2 IPN—LED & MAT—Instituto Pedro Nunes, Laboratory of Tests, Wear and Materials, Rua Pedro Nunes, 3030-199 Coimbra, Portugal; sandracruz@ipn.pt; 3 CEMMPRE—Centre for Mechanical Engineering, Materials and Processes, Chemical Engineering Department, University of Coimbra, 3030-790 Coimbra, Portugal; jcoelho3@gmail.com

**Keywords:** poly(lactic acid), compatibilizers, crystallinity degree, mechanical behavior

## Abstract

Poly (lactic acid) (PLA), due to its biodegradability, biocompatibility, and renewability, is one of the most promising biobased polymers for replacing some of the petrol-based materials. Low flexibility of PLA is overcome, by blending it with olefin-based polymers, such as polypropylene (PP). However, the use of compatibilizing agents is required to attain final materials with suitable mechanical properties. Such agents, although essential, can affect PLA structure and, consequently, the mechanical properties of the PLA. To the best of our knowledge, this issue was never studied, and the results can contribute to achieving the best formulations of PLA-based blends according to their final applications. The thermal and mechanical properties of the extruded PLA, with three different commercial compatibilizing agents, were evaluated with the purpose of demonstrating how the compatibilizers can introduce structural differences into the PLA chain during the extrusion process. The combination of crystallinity, molecular weight, and the morphology of the samples after extrusion determines the final mechanical properties of PLA. Despite being a fundamental study, it is our aim to contribute to the sustainability of PLA-based industries. The addition of a 2.5% concentration of C1 compatibilizer seems to have less influence on the final morphology and mechanical properties of the blends.

## 1. Introduction

Today, our lifestyle relies on polymers and almost all transforming industries need it, from automobile to aeronautic, passing through clothing and food packaging [1]. The vast majority of these polymers are mostly produced from fossil feedstocks and after the normal lifespan of these materials, the majority will be part of the massive pile of non-degradable waste dumps [2]. This led to global concerns about environmental consequences. In 2018, the annual global production of plastic products was around 359 million metric tons, with 62 million metric tons being produced just in Europe [3], and it is expected to increase since these polymers tend to replace other conventional materials such as glass or metals in many less demanding applications. The concern about the sustainability issues associated with petroleum-based polymers [4] has contributed to the development of environmentally friendly polymers that are entirely degradable under composting conditions. Poly (lactic acid) (PLA) is the most successful example of such a type of material that can be used to replace synthetic polymers [5]. Several reviews have been published that discuss the main research topics and also the industrial applications of this polymer [6,7,8,9,10,11,12,13]. In 2018, PLA represented 10.3% of the worldwide production of bioplastics and its production capacities are predicted to double by 2023 [14]—its high production is explained by its high versatility, since PLA can replace several petroleum-based polymers. Moreover, the biodegradation of such a polymer has attracted the attention of the scientific community in recent years, since its application has been growing [15,16,17,18].

PLA is eco-friendly [19], biocompatible [20] and has good thermal processability, however, this biopolymer has some important issues that still limit a broader application, such as lack of reactive side-chain groups [9], hydrophobicity [21,22] and poor toughness [23,24,25,26]. The latter is recognized as the major limitation associated with the materials based on PLA. Additionally, PLA is well known to have low melt strength, which is a drawback [27], especially for the melt-extrusion process. To overcome these problems, plasticizers and low glass transition (T_g_) polymers can be added to PLA formulations to enhance the ability of these polymers to absorb energy and deform plastically without fracturing [28,29,30,31,32]. Commercial PLA Ingeo^®^ was the first synthetic polymer based on renewable sources produced at an industrial scale. However, in most markets, a complete substitution for a biopolymer is difficult, hence the incorporation of PS, PET, and PP, among others. PP is a thermoplastic, characterized by good mechanical and biological properties, chemical resistance, and inertness [33], largely used in an industrial scale. The problem associated with this strategy deals with the much lower mechanical properties of the blends compared with the PP counterpart [34]. Knowing the PLA biodegradability, the preparation of blends of PLA and PP may be a suitable strategy to afford materials with interesting mechanical properties and accelerated decomposition [35]. However, PLA and PP are partially immiscible and constitute multiphase blends with poor mechanical performance. This issue can be mitigated with the addition of compatibilizing agents.

Compatibilizers generally present a macromolecular structure showing interfacial activities in heterogeneous polymer blends. Usually, their structures are composed of block or graft copolymers that are miscible with the components of the blend that, in turn, have active groups formed in situ during the melt-blending process. The compatibilizers are known to reduce the interfacial tension between polymers and increase the interface adhesion between the immiscible phases of the components of blends [2,36]. Good interfacial adhesion is essential for transfer the stress from one phase to the other, thus increasing the efficiency and preventing the cracks, initiated at the interface, from growing until failure. The stabilization of the phase morphology and the enhancement of the interfacial adhesion transform a useless immiscible polymer blend into a material with interesting properties [36,37]. In addition, they can be used as a plasticizer to confer a uniform dispersion of components and to plasticize or soften one of the polymers present in the mixture [38,39].

There are several types of processing technologies for polymers, with extrusion being the most used in the plastic industry. The combination of temperature with screw extruders can melt the polymeric material and provide the mechanical work required over the material [40]. The quality of extruded polymeric materials is highly dependent on the homogeneity of the molten polymer. With mixtures of polymers, the melt state and the work done on mixing are crucial to achieve a homogeneous final material [41].

The melt viscosity of PLA has a stronger dependence on the temperature than PP, which makes control of this processing parameter crucial [42]. This feature is particularly relevant considering the known thermal degradation of PLA at high processing temperatures. Other factors, such as the presence of impurities, residual monomers or residual catalysts, can also enhance the thermal degradation of this polymer. During the production of yarns based on PLA by extrusion at high temperatures, undesired molecular weight reduction and weight loss occur [43].

Since the mixture implies miscibility between them, morphological stability must be observed in both phases. In the particular case of blends of PP with PLA, some studies were developed concerning the structural and morphological changes in the PP when in the presence of a compatibilizer [36,37,44], but, so far, no studies were performed regarding PLA. In this work, it is our aim to fill the gap in the literature regarding the impact of the compatibilizer on the PLA structure. The results may contribute to a more realistic choice of the compatibilizers, but the outputs of this study must be balanced, with the changes promoted by the same compatibilizer on PP structure and morphology.

The thermal and mechanical behavior of PLA was widely studied, but to the best of our knowledge, the effect of the addition of small quantities of compatibilizers on PLA structure has never been reported. Here, we describe a degradation study of PLA in the presence of known PP compatibilizers, making it possible to understand the importance of the type of compatibilizer and respective amounts to use in the melt-extrusion of PLA/PP/compatibilizer. The final materials were studied by size-exclusion chromatography (SEC) and thermal degradation was analyzed by thermogravimetric analysis (TGA). Crystallization studies of the materials were studied using differential scanning calorimetry (DSC). Finally, the mechanical properties were evaluated. A chart diagram, included in Supporting Information (Figure S1), can elucidate the flow of the experimental procedure.

## 2. Materials and Methods

### 2.1. Materials

**Figure 1:**
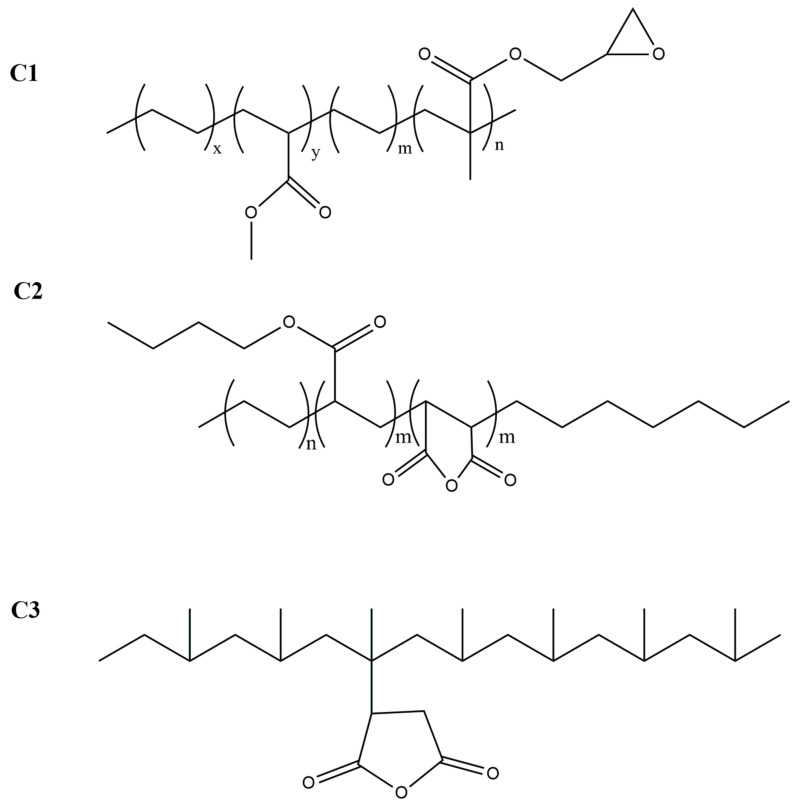
C1, C2 and C3 compatibilizer structures used in this work.

PLA used in this work was of a commercial-grade and was supplied by NatureWorks^®^ LLC (Ingeo^TM^ 2500HP, NatureWorks^®^ LLC, Minnetonka, MN, USA). According to the data supplier datasheets [45], glass transition temperature *T*_g_ is about 55–60 °C, melting temperature *T_m_* is about 150–180 °C, and decomposition temperature is 250 °C. For melt processing, temperatures between 190 °C and 210 °C are recommended. In this work, three different compatibilizers, named C1, C2, and C3, were used (Figure 1).

### 2.2. Extrusion of PLA/Compatibilizer Mixtures

Before the processing, PLA was dried at 50 °C overnight. This step was performed to prevent PLA hydrolysis and possible lactide reformation during extrusion. The PLA pellets were extruded in a laboratory extruder (homemade), with a Length/diameter (L/D) screw ratio from 27:1 originating yarns of diameters between 0.80 mm and 1.30 mm. For mechanical tests, yarns with less diameter variations were selected. About 2.5% (*w*/*w*) and 5% (*w*/*w*) of the different compatibilizers were added to PLA pellets and the mixtures extruded immediately. The screw rotation speed used in all samples was 25 rpm and two processing temperatures ranges were selected: 190–200 °C and 210–220 °C.

### 2.3. Physicochemical Characterization

Proton nuclear magnetic resonance (^1^H NMR) spectra were obtained at room temperature on a Bruker Avance III 400 MHz Spectrometer (Brucker, Ettlingen, Germany) using a 5 mm TIX triple resonance detection probe, in deuterated chloroform (CDCl_3_). Chemical shifts are reported relative to chloroform (δ = 7.26 ppm). The integration of the signals was made by using MestRenova software version 6.0.2-5475.

The morphology of the yarns was assessed by scanning electron microscopy (SEM, CARL ZEISS: MERLIN™, Göttingen, Germany). The specimens were frozen in liquid nitrogen prior to fracture to diminish the risk of plastic deformation. The fractured surfaces were coated with gold and analyzed using a field emission scanning electron microscope (FESEM, CARL ZEISS: MERLIN™, Oberkochen, Germany), Zeiss Merlin Compact/VP Compact, operating at 1 kV.

### 2.4. Thermal Analysis

The thermal stability of the processed PLA and compatibilizers was evaluated in the range of ca. 25–600 °C, in a TA Instruments Q500 thermogravimetric analyzer (Mettler Toledo, Giessen, Germany) (thermobalance sensitivity: 0.1 µg) at a heating rate of 10 °C·min^−1^ and under a dry nitrogen purge flow of 100 mL·min^−1^. The differential scanning calorimetry (DSC) studies were performed within a temperature interval ranging from −10 to 250 °C, in a TA Q100 instrument (Mettler Toledo, Giessen, Germany) at a heating rate of 2 °C·min^−1^ under a nitrogen flow of 50 mL·min^−1^. The crystallinity content (X_c_) has been evaluated from the DSC data according to the following equation [46]:

(1) $$X_{c}\left(\%\right)=100\left(\Delta H_{m}-\Delta H_{c}\right)/\Delta H_{m}^{{}^{\circ}}$$

where Δ*H_m_* and Δ*H_c_* are the melting and the crystallization enthalpies, respectively, and Δ*H°_m_* is the reference Δ*H_m_* (93.6 J/g) for PLA crystals having an infinite size.

### 2.5. Size Exclusion Chromatography (SEC)

The samples were analyzed by a size exclusion chromatography (SEC) system (Viscotek TDAmax, Malvern Instruments Limited, Worcestershire, UK) equipped with an online degasser, and a set of columns comprising a HFIP (hexafluoroisopropanol) guard column followed by two PL HFIPgel (6 μm) columns. The HPLC dual-piston pump was set with a flow rate of 1 mL·min^−1^. The eluent (chloroform) was previously filtered through a 0.2 μm filter. The tests were done at 40 °C using a Perkin Elmer LC Oven 101 heater (Perkin Elmer, Waltham, MA, USA). Before the injection (50 μL), the samples were filtered through a polytetrafluoroethylene (PTFE) membrane with 0.2 μm pore. Polymer molecular weights and polydispersity (M_w_/M_n_) were determined by conventional calibration using Clarity software version 2.8.2.648 (Clarity Software Group, Solihull, UK).

### 2.6. Mechanical Testing Measurements

Tensile testing measurements were performed by using a Chatillon TCD 1000 mechanical tester (CHATILLON, New York, NY, USA), following an adapted procedure of the ISO 527-1:1993 (E), at a speed rate of 10 mm·min^−1^ until fracture. All mechanical data were the average value of, at least, five valid repeated tests. The yarns’ diameters tested in tensile measurements were the average of three different sectors.

## 3. Results and Discussion

### 3.1. Structural Analysis of the Extruded Materials

The extruded mixtures of PLA/compatibilizer were analyzed by ^1^H NMR to detect any signal of degradation and, if so, to identify the presence of unexpected compounds (see Figure S2 Supplementary Materials). Aiming to detect traces of degradation, PLA and PLA + 2.5% compatibilizers, extruded at different temperature ranges (190–200 °C and 210–220 °C), were analyzed. It was not possible to detect any visible differences between the two ranges of temperatures used in PLA processing or even between the compatibilizers C1, C2, and C3. The peaks ascribed to C-H at 5.19, 5.17, 5.15 and 5.13 ppm and to CH_3_ (δ = 1.59; 1.57 ppm) were identified, independently of the compatibilizer used. In addition, the different temperature ranges of processing did not result in detectable degradation products by ^1^H NMR.

**Figure 2:**
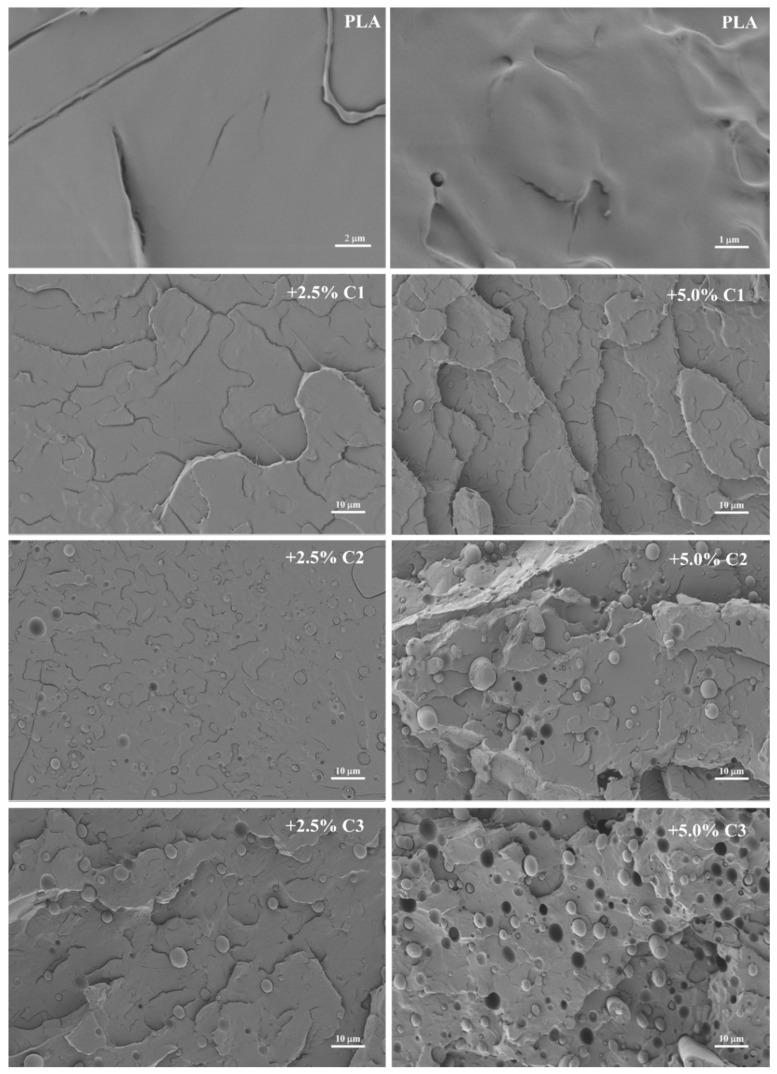
SEM micrographs of PLA (two magnifications, 5000× and 10,000×) and PLA + Compatibilizers (2.5 and 5.0%) magnified 1000× *g*.

The microscopic structure of the processed polymers analysed by SEM showed some significant differences depending on the compatibilizer and its content in the mixture (Figure 2). PLA has a smooth fractured surface whereas the introduction of compatibilizer enhances surface roughness. The appearance of some holes and drops with the introduction of C2 and C3 compatibilizers was also observed. This means that these compatibilizers showed distinct phase formation in the presence of PLA. The compatibilizer C1 (glycidyl based) is the one that causes minor roughness on the polymeric surface. Moreover, increasing the amount of compatibilizers resulted in higher changes in PLA structure.

SEC measurements of the materials were carried out to evaluate possible degradations of PLA polymeric chains. PLA with compatibilizers, having different concentrations (2.5% and 5%) and two processing temperatures, were analyzed. With the same purpose, non-processed PLA was also tested at high and low extrusion temperatures. The temperature ranges, molecular weights, and polydispersity of the mixtures of PLA/compatibilizers are displayed in Table 1.

According to Table 1, a small decrease in the initial molecular weight (M_n_ = 138,000) was observed after processing of PLA, at both ranges of temperature, being more evident, as expected, at higher temperatures (M_n_ = 132,000 for 190–200 °C and M_n_ = 125,000 for 210–220 °C). Although not shown here, for the lower range of processing temperatures, no relevant differences between the types of compatibilizers used were detected. At higher processing temperatures, a decrease in M_n_ for all the samples was observed. This phenomenon is explained by the scission of the PLA chains during the extrusion process. The outcome of increasing the amount of compatibilizer in PLA processing (5%) resulted in higher M_n_ than those obtained only with 2.5%. This fact could be due to the reaction of the terminal groups of PLA (hydroxyl) of different chains with the reactive groups of compatibilizers (epoxide or anhydride) in a similar way as a chain extender. This function is proportional to the amount of material and more probable when a higher amount is present.

### 3.2. Thermal Properties

#### 3.2.1. Termogravimetric Analysis

**Figure 3:**
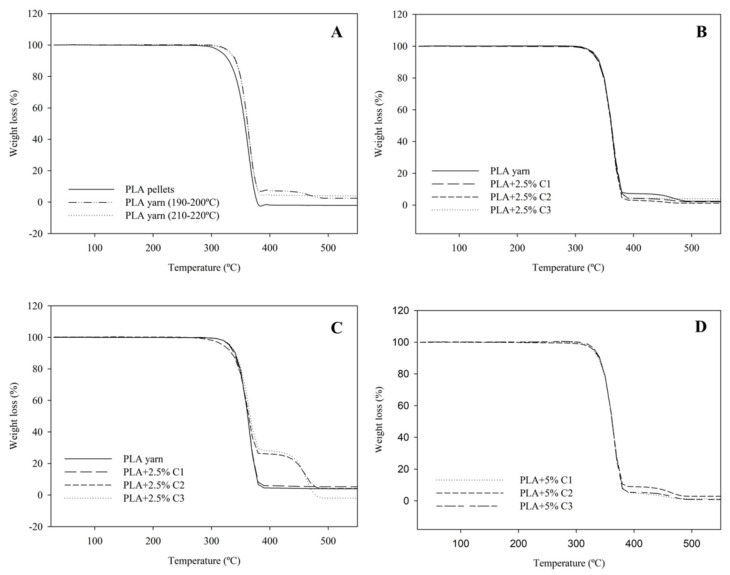
Thermogravimetric (TG) curves at a heating rate of 10 °C·min^−1^ of (**A**) PLA pellets and PLA processed at two different temperature ranges; (**B**) PLA + 2.5% compatibilizers processed at 190–200 °C; (**C**) PLA + 2.5% compatibilizers processed at 210–220 °C and (**D**) PLA + 5% compatibilizers processed at 210–220 °C.

The thermal profile of the processed PLA + compatibilizers was studied and the results are summarized in Figure 3 and Table 2. The mixture of PLA and compatibilizers showed a similar degradation profile, with T_on_ higher than 340 °C. In most cases, the processed PLA showed a single step of weight loss. In addition, T_5%_ and T_10%_ are similar for all the analyzed materials. However, PLA + 2.5%C2 processed at 210–220 °C, and PLA + 2.5%C3 processed at both processing temperature ranges, presented different thermal behaviors. According to Figure 3C,D, these three samples showed two T_on_. Despite these differences in thermal profile, no relevant changes in the thermal stability of PLA were observed when adding compatibilizers C1, C2, and C3. However, PLA + 2.5%C2 processed at both temperature ranges show higher differences when compared with PLA processed at the same temperatures, which are in agreement to the results obtained on M_n_, where C2 showed the lowest values.

In addition, it is worth mentioning that PLA yarn showed a higher initial degradation temperature than the non-extruded PLA. We expected that PLA yarn, due to thermal degradation resulting from the extrusion process, would start to degrade earlier, since the number of short polymer chains increased [47]. This subtle shift suggests that the thermal degradation profile of PLA is not significantly affected by the polymer degradation occurring during the extrusion process [48] and that the possible increase in the oligomers’ presence does not affect the thermal profile of the PLA yarn. This is corroborated by the similar T_on_ of both samples (340 and 346 °C for PLA pellets and extruded PLA, respectively).

#### 3.2.2. Influence of Temperature Processing on PLA Crystallization

The properties of the final product are strongly dependent on the final polymer’s structure and, concerning PLA, by the crystallization process. In this case, after extrusion the material is cooled immediately (as used in usual extrusion and injection molding processes), being the crystallization of PLA mixtures a process too slow to have significant development. Due to the fast cooling process, samples of PLA with high crystallinity is a difficult goal to achieve [49]. PLA samples were analysed by DSC (Figure S3 and Table 3 and Table 4).

The glass transition *T_g_* of unprocessed PLA pellets occurs at 61.2 °C and the melting temperature *T_m_* is found at 177.6 °C, as displayed in Table 3. Both values are close to the supplier data (T*_g_* = 55–60 °C and T*_m_*= 165–180 °C). From these data, the polymer must be mainly composed of the *L* isomer and only a few percent of the *D*,*L* isomer (less than 5%) [50]. The melting enthalpy, ΔH_m_, is estimated at 47.6 kJ/kg, corresponding to the crystallinity of 30.6%, assuming that the melting enthalpy of 100% crystalline polymer is 93.6 kJ/kg [51].

The influence of the processing temperatures and the presence of compatibilizer on PLA characteristics can be analyzed from the DSC results of the different heating cycles (Table 3 and Table 4). Concerning the different extrusion temperatures, the thermal behavior of the PLA yarn does not change significantly, as shown in Figure S4 (see Supporting Information).

According to Table 3 and Table 4, it was possible to observe that the amount of crystalline PLA in the original PLA (pellets) diminished for the second heating (19.6%) compared with the first heating (30.7%). The reason for these differences could be related to the more favorable condition for pristine PLA crystallization during its synthesis than after extruding processes [46]. The DSC study corresponding to the 2nd heating run of the extruded PLA in the presence and absence of compatibilizers in two operational extrusion temperatures is shown in Figure S4 and the results are summarized in Table 4.

**Figure 4:**
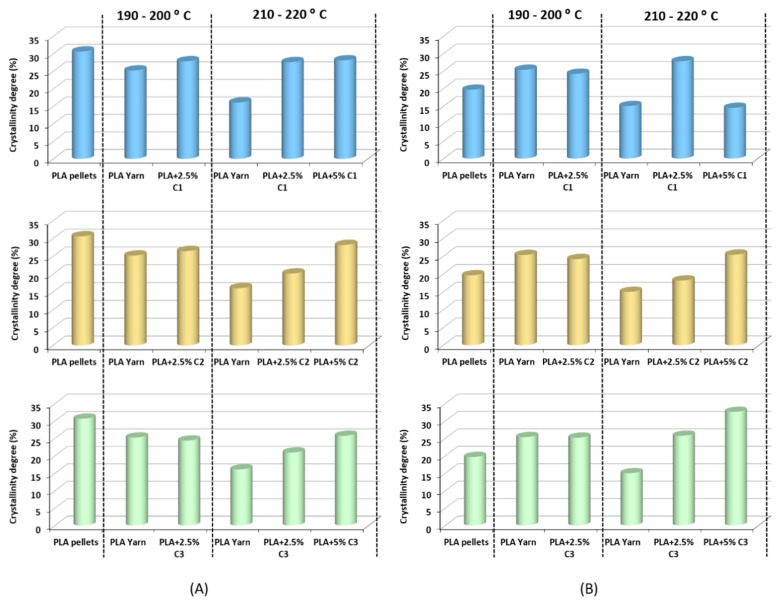
Influence of processing temperatures and types of compatibilizers on the crystallinity degree of the PLA-based samples from the (**A**) first heating cycle and (**B**) second heating cycle.

The values obtained from the second heating erase some uncertainties from the thermal history of the different samples that could cause misleading conclusions [52]. The effect of the processing is the increase in the T*_g_* of the pure PLA from 61.4 °C in the pellet form to 63.8 °C and 64.0 °C in the material processed at 190–200 °C or 210–220 °C extrusion temperature, respectively. Both T*_c_* and T*_m_* showed a small temperature increase due to processing. This fact could be related to some induced crystallization by the extrusion process, as noticed by the supplier of this specific PLA. Analyzing the effect of the different compatibilizers, C1, C2 and C3, on the thermal properties of the extruded PLA, we noticed that at an extruding temperature of 190–200 °C there is no important effect on T*_g_*, T*_c_* or T*_m_* temperatures compared with PLA without compatibilizers processed under the same conditions. In addition, X_c_ showed small differences between the studied samples. However, using a high temperature of extrusion (210–220 °C), some differences occur. For 2.5% and 5% compatibilizer, the T*_g_*, T*_c_* and T*_m_* of the extruded materials are generally lower than those observed for the processed PLA. The ΔH_c_ and the ΔH_m_ in the presence of compatibilizers are also consistently higher than the PLA without compatibilizer. Through the analysis of the degree of crystallinity, different behaviors are verified. Concerning the PLA yarn, at high processing temperatures, the degree of crystallinity decreases in both heating cycles, thus increasing the amount of amorphous phase compared with the polymer processed at a lower temperature. The extrusion process implies that the hot mixture is suddenly cooled with water, and so the amount of amorphous phase is more important for mixtures processed at higher temperatures. The crystallinity degree increases from the samples with 2.5% compatibilizer to 5% compatibilizer, with C1 being the exception to this trend (Table 4 and Figure 4). These facts can be explained by increasing the number of short polymer chains (due to high level of PLA degradation, as observed in the SEC studies) that contributes to a high tendency to crystallization of PLA chains. Compatibilizer C3 appears to be the one that causes higher crystallinity of the samples; this fact is due to its greater tendency to originate PLA with less molecular weight (see SEC results). The exception of C1, in the second heating cycle, can also be related to the molecular weight. Adding 2.5% C1 leads to a dramatic decrease in the molecular weight compared to the PLA yarn, whereas, when adding 5.0%, the compound returns to a molecular weight closer to values of the polymer extruded without compatibilizer. The biggest chains need more energy to reorganize the structure, as proven by the higher amount of the ΔHc.

### 3.3. Mechanical Properties

**Figure 5:**
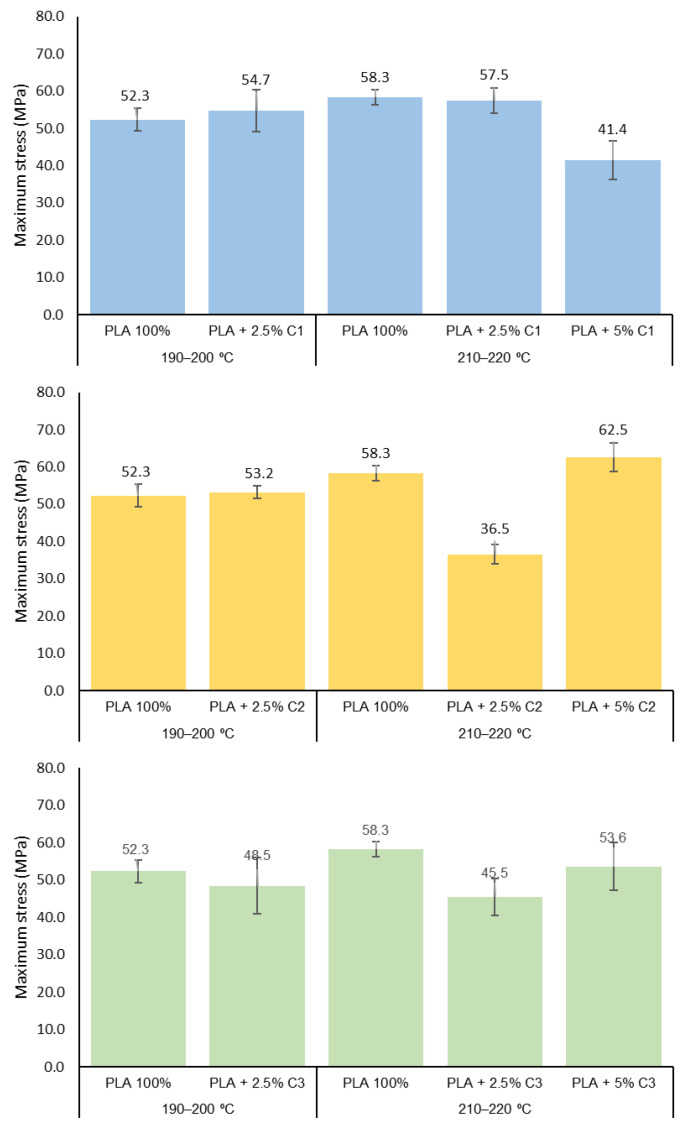
The maximum stress of PLA 100%, PLA + 2.5% and PLA + 5% concentrations of compatibilizer samples extruded at 190–200 °C and 210–220 °C.

**Figure 6:**
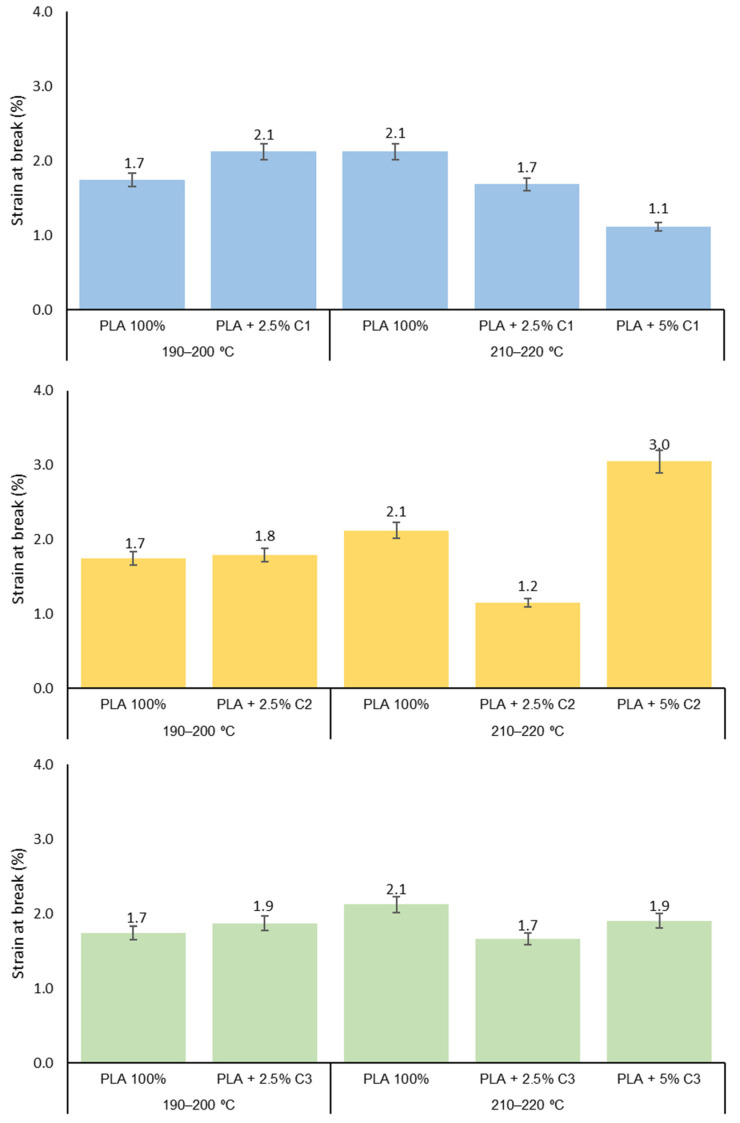
Strain at break of PLA 100%, PLA + 2.5% and PLA + 5% concentrations of compatibilizer samples extruded at 190–200 °C and 210–220 °C.

**Figure 7:**
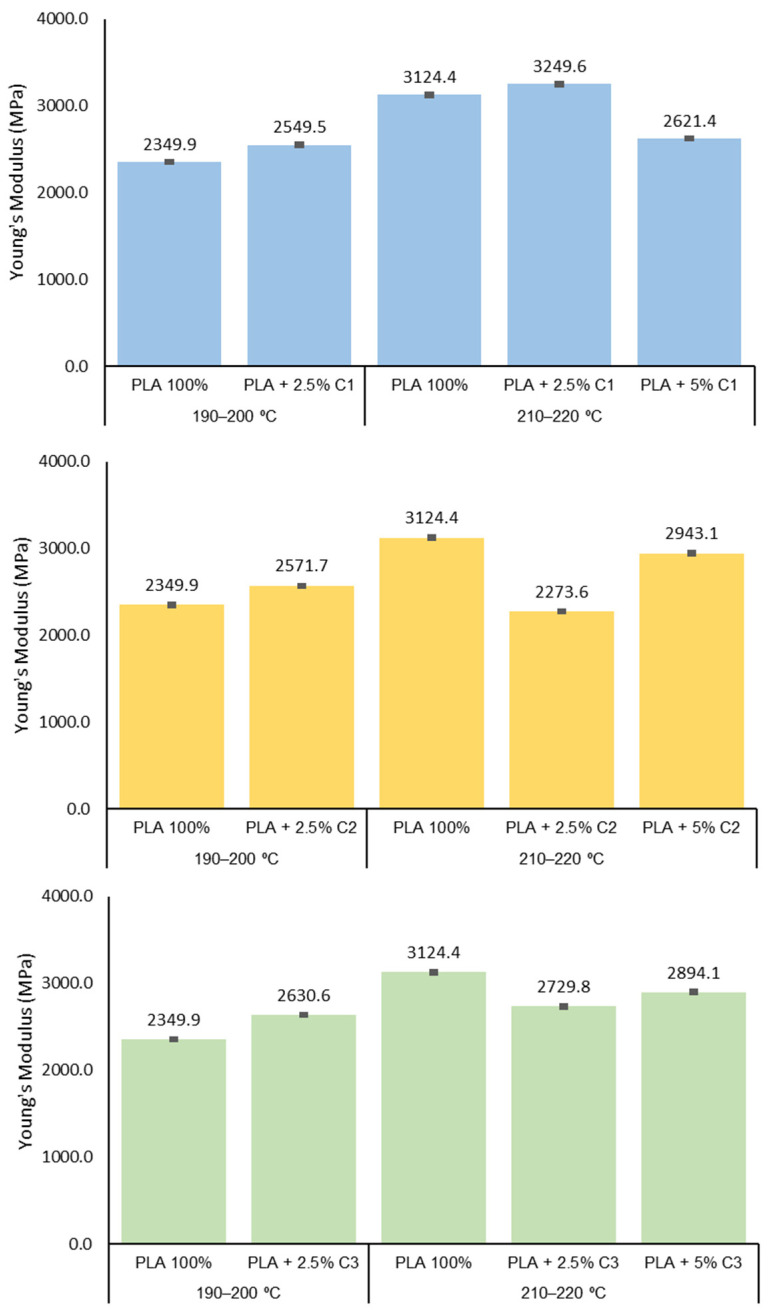
Young’s Modulus of PLA 100%, PLA + 2.5% and PLA + 5% concentrations of compatibilizer samples extruded at 190–200 °C and 210–220 °C.

The effect of compatibilizers on the PLA extrusion, particularly on the crystallization properties of the final material, could be expressed in the final mechanical behavior. The study of the mechanical properties of the PLA yarns extruded with or without compatibilizers and at different extrusion conditions is shown in Figure 5 (maximum stress), Figure 6 (strain at break) and Figure 7 (Young’s modulus).

According to Figure 5, extrusion at the temperature range of 190–200 °C, with 2.5% concentrations of compatibilizers C1, C2 or C3, originate PLA yarns that do not significantly differ in terms of mechanical properties compared to the original PLA. This is in good agreement with the apparent absence of effects over T*_g_*, T*_c_* and T*_m_* observed in the extruded products in this condition. A different situation occurs in the case of PLA extruded at higher temperature (210–220 °C) in the presence of compatibilizers. Overall, a reduction in the mechanical properties is observed.

Looking for the different compatibilizers, in the case of C1, the samples extruded with a 5% concentration of this compound bear smaller maximum stress and lower values of strain at break (Figure 5 and Figure 6). Since the mixture of PLA + 5%C1 shows smaller degradation than the samples with 2.5% C1, the presence of bigger polymeric chains promotes better behavior of the strain of the material.

Regarding the compatibilizer C2, this agent is the one that introduces major changes to the mechanical properties of PLA-based yarns. The samples using concentrations of 2.5% of this compatibilizer show smaller maximum stress and elongation than the sample with only PLA; this result is in agreement with those thermal properties, and since PLA + 2.5%C2 (210–220 °C) has a similar X_C_ of PLA, its mechanical properties are lower than PLA + 5%C2. These properties are recovered when the C2 concentration increases to 5% (Figure 5 and Figure 6). The observed increases in M_n_ and crystallinity degree as the C2 concentration increases contribute to the observed results.

The X_C_ of PLA + 5% C3 is the highest of all the samples studied and this sample presents smaller polymeric chains when processed in the 210–220 °C range. For the C3 compatibilizer, a smaller decrease in all mechanical properties is observed (more evident for the 2.5% concentration), probably due to the reduction in the M_n_ observed for the extruded PLA. The higher crystallinity of the sample with a high concentration of compatibilizer (5%) could explain the increase in the maximum stress and Young’s *modulus* of the 5% sample (Figure 7). The Student’s-t test was used to determine the statistical significance between the PLA samples for maximum strength, strain at break and Young’s modulus (Figure 5, Figure 6 and Figure 7). Concerning the maximum stress of the PLA samples, the addition of 2.5% C1 to the extruded PLA, at both extrusion temperatures, was not considered to be statistically significant (*p* = 0.4289 and *p* = 0.6663, respectively). However, the addition of 5% C1 at PLA is extremely statistically significant (*p* = 0.0004), which is also the case for the other compatibilizers at 5%. On the other hand, for strain at break and Young’s modulus, using a 95% confidence level, the *p*-values were less than 0.0001 (Table S1 to Table S3).

### 3.4. Influence of Compatibilizers Type on PLA Structure

**Figure 8:**
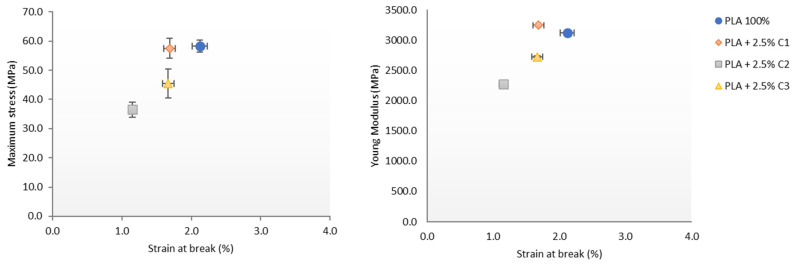
(**Left**) Maximum stress vs. strain at break and (**Right**) Young’s Modulus vs. strain at break, for PLA with 2.5% concentrations of the different compatibilizers.

In order to evaluate, with more precision, the influence of the type of compatibilizer in PLA properties, the tensile tests results were collected and the most important properties presented in Figure 8. The proximity of the main properties, corresponding to the PLA sample with compatibilizer C1 of PLA, is quite clear, considering their maximum stress and strain at break (Figure 8 Left). The same result was observed for the analysis with Young’s modulus and strain at break, where C1 appears to be the compatibilizer with the least deleterious effect over the PLA properties (Figure 8 Right). Considering both of the analyses in Figure 8, C1 is clearly the compatibilizer that has the best affinity with PLA, while C2 appears to be the compatibilizer that induced properties most distant to the pristine PLA. A similar plot of the values for the same properties, with 5% concentrations of the compatibilizers, is presented in the Supporting Information (Figure S5).

## 4. Conclusions

The effect of the extruded PLA, in the presence of commercial compatibilizers in 2.5% and 5.0% amounts, was studied. ^1^H NMR analyses of extruded PLA samples with compatibilizers show no signals beyond those expected from PLA. SEC analyses showed that the presence of compatibilizers in PLA extrusion contributes to molecular weight reduction compared with the original PLA. DSC analyses showed small differences with respect to T_g_ and T_m_. Concerning crystallinity, some differences were observed in the second heating run, probably due to the presence of small PLA chains. Regarding the mechanical properties, at low processing temperatures, the addition of a 2.5% concentration of compatibilizers does not show significant changes relative to pure PLA. At higher temperatures of extrusion, the three compatibilizers show a particular mechanical behavior, varying with the concentration introduced to the PLA blends. In the case of C1, the mechanical properties’ values decrease as their content increases. For compatibilizers C2 and C3, their trends are similar for all mechanical properties tested, with the mechanical behavior increasing as the content increases. However, the majority of such values are above the PLA ones, which may indicate that the maleic anhydride, present in both compositions, may be responsible for the main changes in the mechanical properties of PLA after the processing mechanism. The morphological analysis of the PLA and PLA with the different compatibilizers, at 2.5 and 5% concentrations, showed distinct results. The addition of both amounts of compatibilizers C2 and C3 resulted in significant changes in PLA morphology (as seen in SEM), corresponding to the decrease in mechanical properties, particularly at 2.5%. On the contrary, the addition of C1, at the amount of 2.5%, did not significantly alter the PLA morphology, which can be related to the results obtained, and did not affect the tensile properties, since the maximum stress was similar to the PLA and the Young’s modulus was higher.

## Figures and Tables

**Table 1:** Molecular weight and polydispersity of PLA and PLA/compatibilizer mixtures at different processing temperature ranges. Mn is the molecular weight and PD is the polydispersity.

Samples	Processing T. Range (°C)	M_n_	PD
Pellets PLA Ingeo 2500 HP	Not processed	138,000	1.5
100% PLA	190–200	132,000	1.5
100% PLA	210–220	125,000	1.5
PLA + 2.5%C1		86,800	1.6
PLA + 2.5%C2		65,200	1.6
PLA + 2.5% C3		81,000	1.5
PLA + 5%C1		107,235	1.5
PLA + 5%C2		101,808	1.5
PLA + 5%C3		95,653	1.5

**Table 2:** Degradation temperatures (T_x%_) and onset temperature (T_on_) of extruded PLA samples with or without different compatibilizers.

Samples	Processing T. Range (°C)	T_5%_	T_10%_	T_on_
PLA	190–200	333.9	342.2	346.7
PLA + 2.5%C1		331.1	340.2	346.8
PLA + 2.5%C2		332.4	349.7	346.6
PLA + 2.5%C3		333.5	341.9	347.0; 436.8
100% PLA	210–220	331.8	341.0	346.4
PLA + 2.5%C1		330.8	339.1	346.0
PLA + 2.5%C2		320.7	335.4	342.3; 440.8
PLA + 2.5%C3		333.3	341.4	345.2; 444.6
PLA + 5%C1		329.7	338.6	346.2
PLA + 5%C2		330.9	340.3	346.2
PLA + 5%C3		333.1	341.0	346.2

**Table 3:** Transition temperatures (glass transition temperature T_g_, crystallization temperature T_c_ and melting temperature T_m_), enthalpies and crystallinity degree of the samples after different conditions of processing obtained from the first heating cycle with a rate of 10 °C min^−1^.

Sample	Processing Temperature (°C)	T_g_ (°C)	T_c_ (°C)	T_m_ (°C)	ΔH_c_ (kJ/kg)	ΔH_m_ (kJ/kg)	X_C_ (%)
PLA pellets	-	61.2	101.3	177.6	18.9	47.6	30.7
PLA Yarn	190–200	62.1	97.9	180.0	27.4	51.0	25.2
PLA + 2.5% C1		61.8	96.4	178.1	26.7	52.8	27.9
PLA + 2.5% C2		61.8	97.7	179.6	24.7	49.5	26.5
PLA + 2.5% C3		62.2	97.8	179.7	22.7	45.5	24.4
PLA Yarn	210–220	61.6	98.6	178.4	13.9	28.9	16.1
PLA + 2.5% C1		61.0	98.0	177.3	30.2	56.1	27.6
PLA + 2.5% C2		62.43	97.9	176.3	17.6	36.5	20.2
PLA + 2.5% C3		63.8	98.4	177.0	13.9	33.5	21.0
PLA + 5% C1		61.4	96.8	179.1	24.9	51.2	28.1
PLA + 5% C2		60.8	96.7	178.2	23.6	50.0	28.2
PLA + 5% C3		60.5	96.5	176.7	26.5	50.6	25.8

**Table 4:** Transition temperatures, (glass transition temperature T_g_, crystallization temperature T_c_ and melting temperature T_m_), enthalpies and crystallinity degree of the extruded PLA samples after different conditions of processing obtained from the second heating cycle with a rate of 10 °C/min.

Sample	Processing Temperature (°C)	Tg (°C)	Tc (°C)	Tm (°C)	ΔHc (kJ/kg)	ΔHm (kJ/kg)	X_C_ (%)
PLA pellets	-	61.4	98.1	175.3	28.7	47.0	19.6
PLA Yarn	190–200	63.8	99.8	176.5	26.3	50.0	25.3
PLA + 2.5% C1		64.3	98.6	176.1	31.4	54.1	24.2
PLA + 2.5% C2		64.0	100.5	176.6	28.9	51.6	24.2
PLA + 2.5% C3		64.3	100.0	175.8	25.0	48.5	25.1
PLA Yarn	210–220	64.0	100.3	176.1	15.4	29.4	15.0
PLA + 2.5% C1		63.3	99.8	175.1	31.8	57.8	27.7
PLA + 2.5% C2		62.8	98.6	173.7	19.4	36.4	18.2
PLA + 2.5% C3		63.8	98.4	174.7	15.1	39.1	25.7
PLA + 5% C1		63.8	106.4	175.8	33.3	46.9	14.5
PLA + 5% C2		63.6	100.3	174.9	26.5	50.3	25.4
PLA + 5% C3		63.3	98.9	173.9	23.1	53.5	32.5

## Supplementary Materials

The following are available online at https://www.mdpi.com/2073-4360/12/11/2688/s1, Figure S1. The chart diagram of the experimental procedure; Figure S2. ^1^H NMR spectra of PLA and PLA + 2.5% compatibilizers, processed at 190–200 °C and 210–220 °C. A. PLA processed at 190–200 °C; B. PLA processed at 210–220 °C; C. PLA + 2.5%C1, processed at 190–200 °C; D. PLA + 2.5%C1 processed at 210–220 °C; Figure S3. DSC thermographs of PLA pellets and PLA yarn extruded at different ranges of temperatures (curves correspond to 1st heating); Figure S4. DSC thermographs of PLA yarn extruded with A. compatibilizer C1 (2.5 and 5%); B. compatibilizer C2 (2.5 and 5%) and C. compatibilizer C3 (2.5 and 5%), at different ranges of extrusion temperatures (curves correspond to 2nd heating); Figure S5. (A) Maximum stress vs. strain at break and (B) Young’s Modulus vs. strain at break, for PLA with 5% of the different compatibilizers. Table S1. Results obtained for the t-test using a 95% confidence level (Maximum stress), being df the degree of freedom. Table S2. Results obtained for the t-test using a 95% confidence level (Strain at break), being df the degree of freedom. Table S3. Results obtained for the t-test using a 95% confidence level (Young’s modulus), being df the degree of freedom.
