# Supplementary material for: The Impact of the Addition of Compatibilizers on Poly (lactic acid) (PLA) Properties after Extrusion Process
Source: Polymers (Basel). 2020 Nov 14;12(11):2688. doi: 10.3390/polym12112688 (PMC7697721; doi:10.3390/polym12112688)
Supplement: Supplementary file 1 [file polymers-12-02688-s001.pdf]

Supporting Information

How compatibilizers addition influence the Poly (lactic acid) (PLA) PLA structure in the extrusion process: Thermal and mechanical analysis

F.A.M.M. Gonçalves[1,+], Sandra M. A. Cruz[2,3,+], Jorge F. J. Coelho[3] and Arménio C. Serra[3,*]

[1] CIEPQPF, Department of Chemical Engineering, University of Coimbra, 3030-790 Coimbra, Portugal; filipaalmeidamartins@gmail.com
[2] IPN - LED & MAT - Instituto Pedro Nunes, Laboratory of Tests, Wear and Materials, Rua Pedro Nunes, 3030-199 Coimbra, Portugal; sandracruz@ipn.pt
[3] CEMMPRE - Centre for Mechanical Engineering, Materials and Processes, Chemical Engineering Department, University of Coimbra, 3030-790 Coimbra, Portugal; jcoelho3@gmail.com, armenio.serra@gmail.com
+ These authors contributed equally to this work.
* Correspondence: armenio.serra@gmail.com.

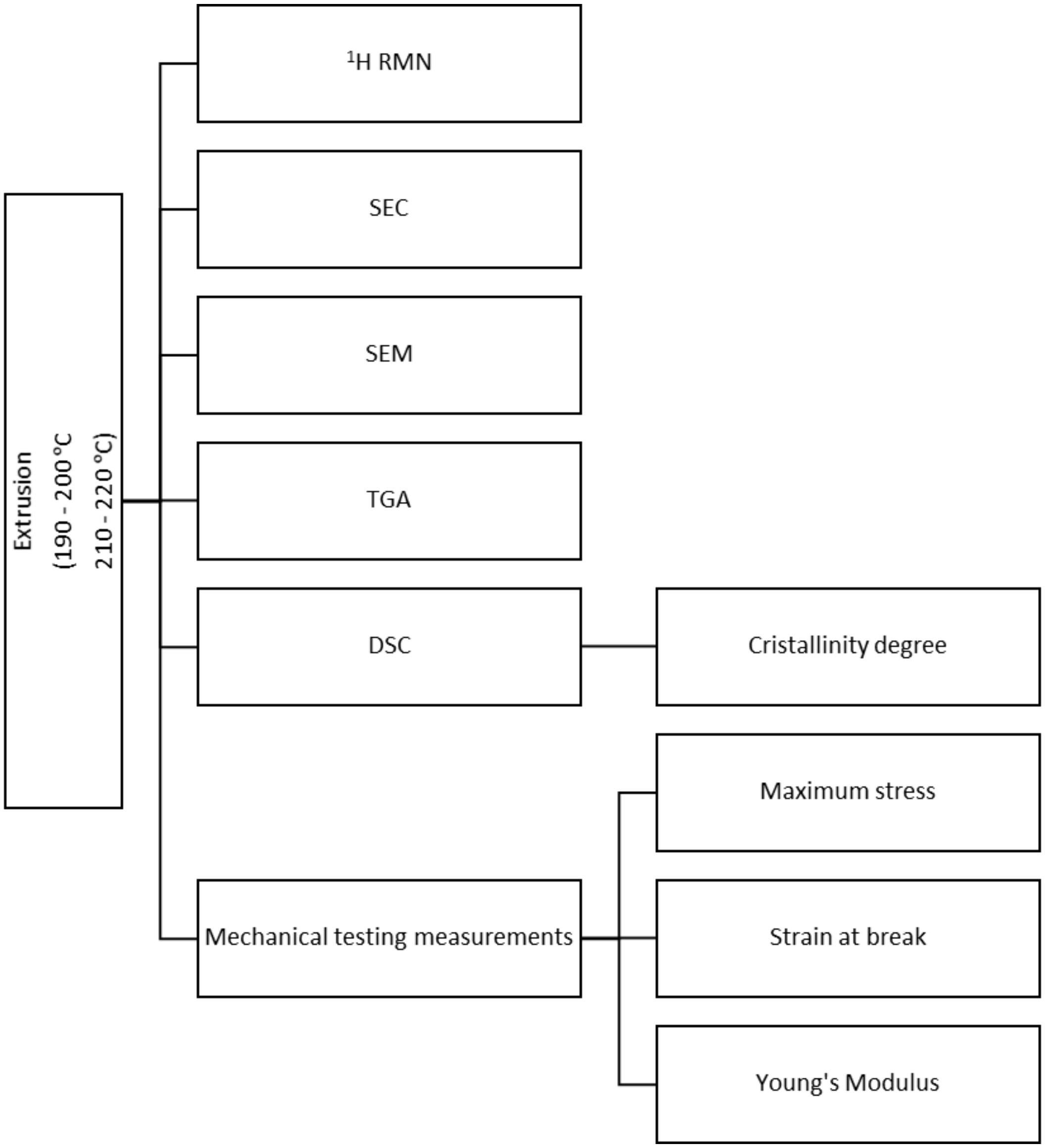

Figure S1. The chart diagram of the experimental procedure.

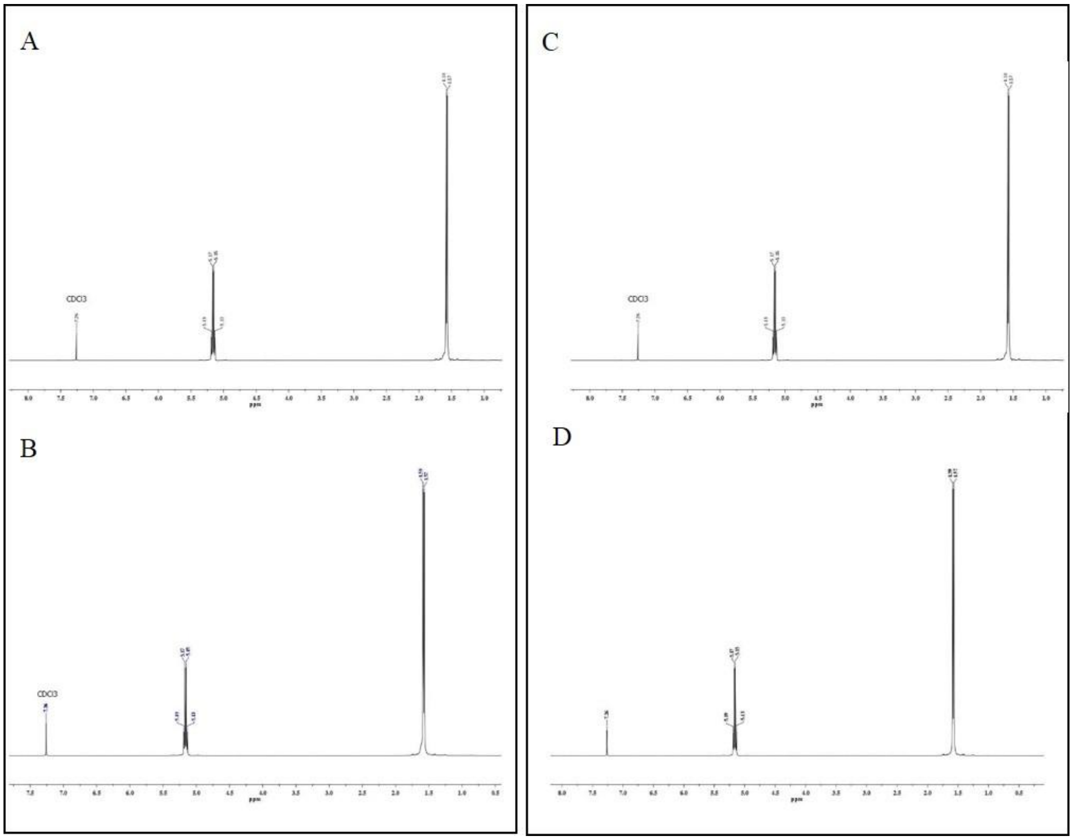

Figure S2. ^{1}H NMR spectra of PLA and PLA+2.5% compatibilizers, processed at 190-200 ºC and 210-220 ºC. **A.** PLA processed at 190-200 ºC; **B.** PLA processed at 210-220 ºC; **C.** PLA+2.5%C1, processed at 190-200 ºC; **D.** PLA+2.5%C1 processed at 210-220 ºC.

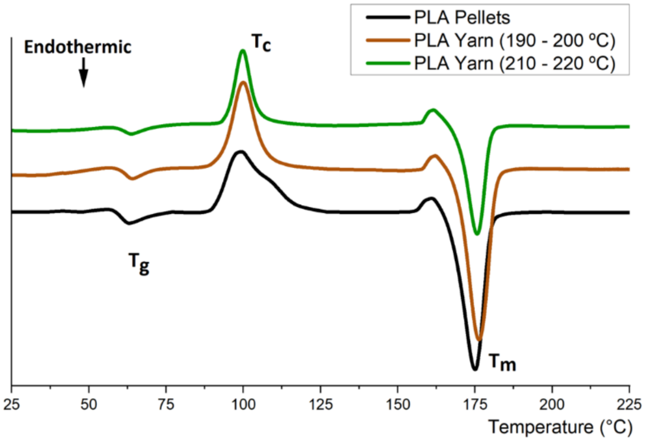

Figure S3. DSC thermographs of PLA pellets and PLA yarn extruded at different ranges of temperatures (curves correspond to 1st heating).

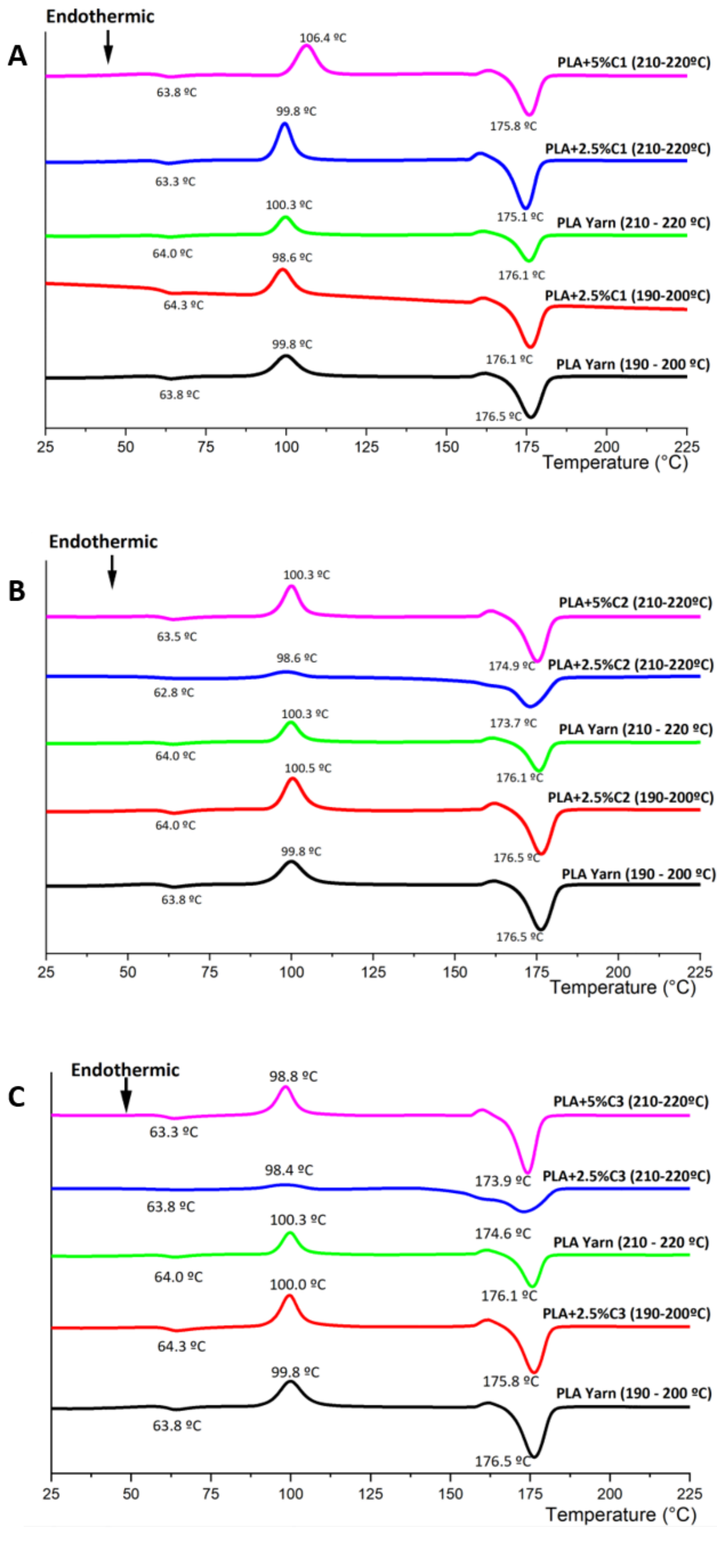

Figure S4. DSC thermographs of PLA yarn extruded with **A.** compatibilizer C1 (2.5 and 5%); **B.** compatibilizer C2 (2.5 and 5%) and **C.** compatibilizer C3 (2.5 and 5%), at different ranges of extrusion temperatures (curves correspond to 2nd heating).

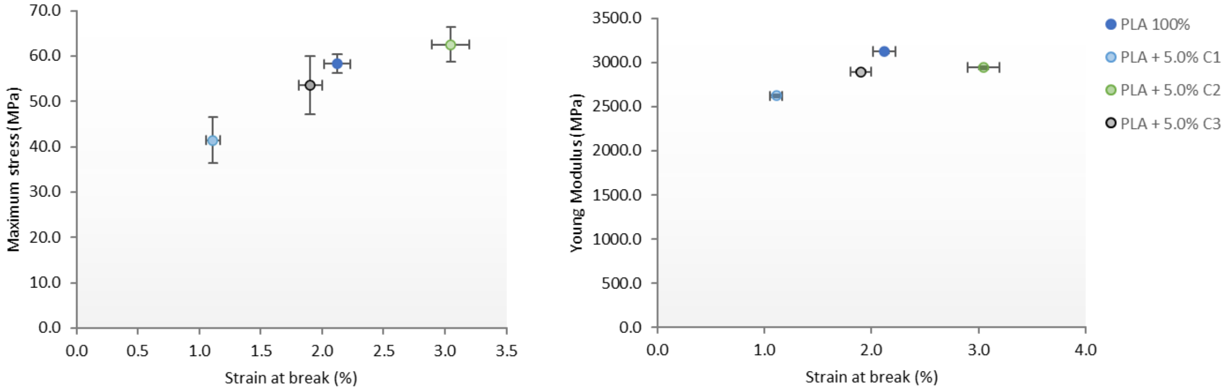

Figure S5. (A) Maximum stress *vs* strain at break and (**B**) Young's Modulus *vs s*train at break, for PLA with 5% of the different compatibilizers.

Table S1. Results obtained for the *t*-test using a 95% confidence level (Maximum stress), being *df* the degree of freedom.

Group	Compatibilizer	PLA samples	t-value	df	p-value
Maximum stress	**C1**	PLA (190 °C)<br>PLA 2.5%C1 (190 °C)	0.8332	8	0.4289
		PLA (210 °C)<br>PLA 2.5%C1 (210 °C)	0.4476		0.6663
		PLA (210 °C)<br>PLA 5%C1 (210 °C)	6.8516		0.0001
		PLA 2.5%C1 (210 °C)<br>PLA 5%C1 (210 °C)	5.8734		0.0004
		PLA 2.5%C1 (190 °C)<br>PLA 2.5%C1 (210 °C)	0.9433		0.3731
		PLA (190 °C)<br>PLA (210 °C)	3.6637		0.0064
	**C2**	PLA (190 °C)<br>PLA 2.5%C2 (190 °C)	0.5836	8	0.5756
		PLA (210 °C)<br>PLA 2.5%C2 (210 °C)	14.5853		<0.0001
		PLA (210 °C)<br>PLA 5%C2 (210 °C)	2.1202		0.0668
		PLA 2.5%C2 (210 °C)<br>PLA 5%C2 (210 °C)	12.4035		<0.0001
		PLA 2.5%C2 (190 °C)<br>PLA 2.5%C2 (210 °C)	12.0209		<0.0001
	**C3**	PLA (190 °C)<br>PLA 2.5%C3 (190 °C)	1.0399	8	0.3288
		PLA (210 °C)<br>PLA 2.5%C3 (210 °C)	5.3689		0.0007
		PLA (210 °C)<br>PLA 5%C3 (210 °C)	1.5603		0.1573
		PLA 2.5%C3 (210 °C)<br>PLA 5%C3(210 °C)	2.2471		0.0548
		PLA 2.5%C3 (190 °C)<br>PLA 2.5%C3 (210 °C)	0.7418		0.4794

Table S2. Results obtained for the *t*-test using a 95% confidence level (Strain at break) , being df the degree of freedom.

Group	Compatibilizer	PLA samples	t-value	df	p-value
Strain at break	**C1**	PLA (190 °C)<br>PLA 2.5%C1 (190 °C)	6.3246	8	0.0002
		PLA (210 °C)<br>PLA 2.5%C1 (210°C)	6.3246		0.0002
		PLA (210 °C)<br>PLA 5%C1 (210°C)	15.8114		<0.0001
		PLA 2.5%C1 (210°C)<br>PLA 5%C1 (210°C)	9.4868		<0.0001
		PLA 2.5%C1 (190 °C)<br>PLA 2.5%C1 (210°C)	6.3246		0.0002
		PLA (190 °C)<br>PLA (210 °C)	6.3246		0.0002
	**C2**	PLA (190 °C)<br>PLA 2.5%C2 (190 °C)	1.5811	8	0.1525
		PLA (210 °C)<br>PLA 2.5%C2 (210°C)	14.2302		<0.0001
		PLA (210 °C)<br>PLA 5%C2 (210°C)	9.0000		<0.0001
		PLA 2.5%C2 (210°C)<br>PLA 5%C2 (210°C)	18.0000		<0.0001
		PLA 2.5%C2 (190 °C)<br>PLA 2.5%C2 (210°C)	9.4868		<0.0001
	**C3**	PLA (190 °C)<br>PLA 2.5%C3 (190 °C)	3.1623	8	0.0133
		PLA (210 °C)<br>PLA 2.5%C3 (210°C)	6.3246		0.0002
		PLA (210 °C)<br>PLA 5%C3 (210°C)	3.1623		0.0133
		PLA 2.5%C3 (210°C)<br>PLA 5%C3(210°C)	3.1623		0.0133
		PLA 2.5%C3 (190 °C)<br>PLA 2.5%C3 (210°C)	3.1623		0.0133

Table S3. Results obtained for the *t*-test using a 95% confidence level (Young's modulus) , being df the degree of freedom.

Group	Compatibilizer	PLA samplea	t-value	df	p-value
Young's modulus	**C1**	PLA (190 °C)	24.5375	8	<0.0001
		PLA 2.5%C1 (190 °C)			
		PLA (210 °C)	12.2574		
		PLA 2.5%C1 (210°C)			
		PLA (210 °C)	52.0508		
		PLA 5%C1 (210°C)			
		PLA 2.5%C1 (210°C)	65.2326		
		PLA 5%C1 (210°C)			
		PLA 2.5%C1 (190 °C)	74.7354		
		PLA 2.5%C1 (210°C)			
		PLA (190 °C)	85.1428		
		PLA (210 °C)			
	**C2**	PLA (190 °C)	27.4885	8	<0.0001
		PLA 2.5%C2 (190 °C)			
		PLA (210 °C)	95.2021		
		PLA 2.5%C2 (210°C)			
		PLA (210 °C)	18.5893		
		PLA 5%C2 (210°C)			
		PLA 2.5%C2 (210°C)	80.0148		
		PLA 5%C2 (210°C)			
		PLA 2.5%C2 (190 °C)	37.7899		
		PLA 2.5%C2 (210°C)			
	**C3**	PLA (190 °C)	32.9925	8	<0.0001
		PLA 2.5%C3 (190 °C)			
		PLA (210 °C)	39.9652		
		PLA 2.5%C3 (210°C)			
		PLA (210 °C)	22.0652		
		PLA 5%C3 (210°C)			
		PLA 2.5%C3 (210°C)	16.3123		
		PLA 5%C3(210°C)			
		PLA 2.5%C3 (190 °C)	10.9190		
		PLA 2.5%C3 (210°C)			
